# Spray Coated Colloidal Quantum Dot Films for Broadband Photodetectors

**DOI:** 10.3390/nano9121738

**Published:** 2019-12-06

**Authors:** Kaixuan Song, Jifeng Yuan, Ting Shen, Jiuyao Du, Ruiqi Guo, Tönu Pullerits, Jianjun Tian

**Affiliations:** 1 Institute for Advanced Materials and Technology, University of Science and Technology Beijing, Beijing 100083, China; songkx1969931415@163.com (K.S.); 13301203163@163.com (J.Y.); shentingustb@163.com (T.S.); jiuyaodu@163.com (J.D.); ruiqiguo1217@163.com (R.G.); 2 Department of Chemical Physics and NanoLund, Lund University, Box 124, 22100 Lund, Sweden; tonu.pullerits@chemphys.lu.se

**Keywords:** quantum dots, spray coating, photodetector

## Abstract

A technique for scalable spray coating of colloidal CdSeTe quantum dots (QDs) for photovoltaics and photodetector applications is presented. A mixture solvent with water and ethanol was introduced to enhance the adhesive force between QDs and the substrate interface. The performance of the detector reached the highest values with 40 spray coating cycles of QD deposition. The photodetectors without bias voltage showed broadband response in the wavelength range of 300–800 nm, and high responsivity of 15 mA/W, detectivity of more than 10^11^ Jones and rise time of 0.04 s. A large size QD-logo pattern film (10 × 10 cm^2^) prepared by the spray coating process displayed excellent uniformity of thickness and absorbance. The large area detectors (the active area 1 cm^2^) showed almost the same performance as the typical laboratory-size ones (the active area 0.1 cm^2^). Our study demonstrates that the spray coating is a very promising film fabrication technology for the industrial-scale production of optoelectronic devices.

## 1. Introduction

Semiconductor nanocrystals with characteristic size below the Bohr radius of the charge carriers are called quantum dots (QDs). Application of the QDs in optoelectronic devices have attracted great attention because of their distinctive physical characteristics, such as size-tunable bandgap, high extinction coefficient, narrow emission spectra, high luminescent efficiency, and multiple exciton generation [1,2,3,4]. Colloidal QDs have been used to construct various optoelectronic devices like solar cell, light-emitting diodes, and photodetectors [5,6,7,8,9]. For example, detectors based on colloidal QDs have advantages like superior response range, high detectivity, low dark current density, high operating temperatures, excellent stability, and weak light response [10]. Photodetectors, which can capture optical signals and convert them into electrical signals, are playing increasingly significant role in many fields like optical communication, environmental monitoring, night vision and biomedical imaging [11,12,13,14]. For example, PbS quantum dots and 2D nonlayered CdS_x_Se_1−x_ nanosheet hybrid photodetectors with a detectivity of up to 1.05 × 10^15^ Jones have been constructed [15]. Zhang et al. fabricated a vertical field-effect phototransistor based on PbS quantum dots, which displayed a high detectivity of 2 × 10^13^ Jones [16]. Peng et al. assembled a flexible photodetector based on the PbS QDs/ZnO nanoparticles with a detectivity of 3.98 × 10^12^ Jones [17]. Both rigid and flexible broadband photodetectors based on CdSeTe colloidal QDs have been made by our group [3,18]. These detectors reached remarkably high detectivity of more than 2 × 10^12^ Jones in the range of 300–800 nm without an external bias voltage [18]. These results demonstrate that QDs based detectors have excellent performance. In addition, colloidal QDs are more suitable for the low-cost, solution-processable fabrication of photodetectors than traditional semiconductor materials because they can be easily deposited on various substrates without the need for high temperature or vacuum [5,19].

Among the deposition methods of colloidal QDs, spin coating technology is usually employed to obtain high-quality QDs-films. Spin coating is a mature technology with the advantages of simple process, high repeatability, and good film quality [20]. However, this process technology faces a great limitation that is only suitable for laboratory scale rather than commercial application due to the small area of the film prepared by spin coating [21,22]. In order to solve such problems, it is necessary to develop a large-scale QDs film preparation process. In our previous work [23], the dip-coating process for QDs film had been investigated, and the QD film photodetector with high detectivity of 4 × 10^12^ Jones was demonstrated. However, dip coating is not suitable as a large-scale manufacturing technology because of low productivity. As an alternative, the spray coating is a very efficient and scalable film manufacturing method. The method is suitable for both hard and flexible substrates. It can be used for preparing thin films on large area substrates. In spray coating the liquid precursor is atomized and ejected by pressurized gas in the spray gun. The droplets fall onto the hot substrate. After the solvent quickly evaporates, the solute deposits on the substrate to form a thin film. The spray coating process has been applied to preparation of active films, such as solar cells [24,25,26,27], light-emitting diodes [28,29], photodetectors [22,30,31,32,33]. Compared with the oxide films deposited by spray pyrolysis, there are still some problems in the preparation of QD films by spray coating. The QDs are dispersed in the solution, and no in-situ reaction is involved in the film-forming process to increase the adhesion between the QDs and the substrate, and only depends on the adsorption of the QDs and the substrate. Therefore, obtaining high-quality colloidal QDs films by spray coating is still a challenge.

In this work, we designed a spray coating platform to prepare the colloidal ternary CdSeTe QD film for the assembly of photodetectors with a response range of 300–850 nm. To balance the solvent volatilization and the adhesive force of the QDs to the substrate, a mixture solvent with water and ethanol was proposed to improve the quality of the thin film. We have investigated the effects of nozzle height, substrate temperature and the number of spray cycles on the film morphology and device performance. The photodetector made by spray-coated QD film reached the best performance when the number of spray cycles is 40. The champion detectors demonstrated responsivity of 15 mA/W, detectivity of more than 10^11^ Jones and rise time of 0.04 s. The large size (10 × 10 cm^2^) QD film pattern prepared by spray coating showed excellent uniformity in absorbance in the ultraviolet-visible spectrum. In addition, we found that the photodetector assembled by large-size QDs film (the active area 1 cm^2^) has almost the same performance as the device of a typical laboratory-size QDs film (the active area 0.1 cm^2^). Successful preparation of large-area QD films for high-performance photodetectors lays foundation for manufacturing of large-scale QD devices.

## 2. Materials and Methods

### 2.1. Chemicals

The main chemicals used are shown in Table 1. The chemicals were used as they are without any further purification.

### 2.2. CdSeTe QD Synthesis

CdSeTe QDs were synthesized according to the previous work with some minor modifications [23]. 0.3084 g CdO, 18 mL paraffin liquid, and 6 mL OA were placed into a 200 mL three-necked flask. 0.0316 g Se, 1 mL TOP and 3 mL paraffin liquid were placed into a 50 mL three-necked flask. 0.026 g Te, 0.5 mL TOP and 1.5 mL paraffin liquid were placed into a 50 mL three-necked flask. These precursors were stirred at room temperature for 30 min while vacuuming, and then the temperature was raised to 60 °C. Ar_2_ was pumped into the flask for two minutes, then the flasks were degassed for 30 min before raising the temperature to 90 °C. If there was no gas in the flasks, Ar_2_ was injected into the flasks until the end of the experiment. The temperature of Cd precursor was eventually set at 260 °C until dissolved. The corresponding temperature for Se precursor is 150 °C, and for Te precursor 300 °C. Finally, each precursor was stored at a temperature of 50 °C and was ready for the next step. The mixed precursors (milliliter ratio, Cd:Se:Te = 5:0.7:0.3) were put into a 100 mL three-necked flask. The mixture was stirred at 50 °C for 5 min while vacuuming. The temperature of the flask was set to 90 °C and Ar_2_ was pumped for 10 min. Then the flask was dried until there was no gas. Ar_2_ was again pumped into the flask and the temperature was raised, the precursor was stirred at a 310 °C for 15 min. Then the flask was cooled to 260 °C, and 4 mL of OAm was rapidly injected into the mixture. Finally, the flask was cooled to room temperature. The QDs capped with OAm were obtained by centrifugation and dispersed in dichloromethane.

### 2.3. Device Fabrication

FTO covered glasses were successively put into ionized water, acetone, and ethanol for ultrasonic cleaning for 20 min, and then treated with UV-ozone for 20 min. A compact TiO_2_ film was deposited on the FTO substrate by spray pyrolysis at 450 °C. When the temperature of FTO was reduced to room temperature, a layer of mesoporous TiO_2_ was spin-coated with 4000 rpm for 30 s, and then heated to 450 °C for 30 min. The ligands of QDs were exchanged by mercapto acetic acid (TGA) to make them water solvable [3]. Then the TGA-QDs were dissolved in a mixture of deionized water and ethanol with a volume ratio of 2:1. QDs Spray Deposition: the distance between the nozzle and the substrate was set to 18 cm, the pressure value of the spray was 0.3 MPa, and the substrate heating temperature was 100 °C. The automatic motor controlled the cycling motion of the spray gun by setting different spray cycle times. The QD film was covered by a layer of Spiro-OMeTAD formed by spin coating with 4000 rpm for 30 s. Finally, thermal evaporation was used to make a layer of the Ag electrode under a vacuum.

### 2.4. Characterization

The scanning electron microscopy (SEM) images were measured by using a scanning electron microscope (SU8020, Hitachi Co., Tokyo, Japan). The UV-vis absorption of QDs and films were measured by an ultraviolet (UV) near-infrared (NIR) spectrophotometer (Puxi-T10, PERSEE, Beijing, China). Photoluminescence (PL) of QDs was measured by the Guangdong luminescence spectrometer FL-380 (GANGDONG, Tianjin, China). The transition electron microscopy (TEM) images were measured by a TEM instrument (JEM-2010, Hitachi Co., Tokyo, Japan). The current density-voltage curves were measured by a Keithley 2400 multimeter (TE Connectivity, Schaffhausen, Switzerland) under AM 1.5 G illumination simulated sunlight (100 mW/cm^2^) (7-SS1503A). The structure of the materials was measured by X-ray diffraction (XRD, PANalytical B.V., Almelo, Netherlands) with Cu Kα radiation. The responsivity was measured by a Keithley 2400 multimeter, a 150 W xenon lamp (SOFN, Beijing, China) and a Spectral Product DK240 monochromator (Spectral Products, Putnam, CT, USA). The current-time response was measured by an electrochemical workstation (Chenhua, Shanghai, China).

## 3. Results and Discussion

**Figure 1:**
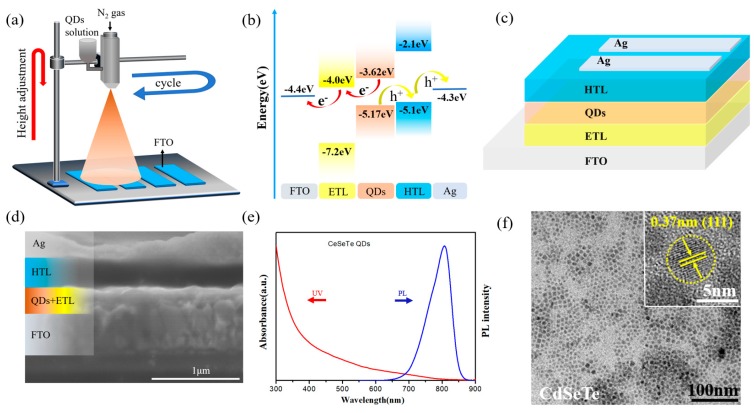
(**a**) Schematic illustration of the spray coating platform. (**b**) Energy band diagram. (**c**) Device structure. (**d**) Cross-sectional SEM image of the photodetector. (**e**) UV-vis absorption and PL spectra of CdSeTe QDs (at 470 nm excitation). (**f**) The TEM image of the CdSeTe QD (inset image is HRTEM).

Figure 1a shows the schematic diagram of the spray coating platform. The air pressure, the distance between nozzle and substrate can be adjusted for processing QDs film. QDs solution will be atomized into liquid mist by a pressurized N_2_ nozzle. When the liquid mist drops onto the substrate while being heated, the solvent will evaporate rapidly. The residual solute QDs condense on the substrate to form a compact film. The surface of colloidal CdSeTe QDs is initially coated with oleylamine (OAm) ligands. Here, in order to improve the charge carrier mobility of QDs film, the OAm ligands are replaced with short-chain mercapto acetic acid (TGA) ligand according to the previous work [3]. Dichloromethane (CH_2_Cl_2_) as the solvent of the QD solution is also exchanged to deionized water. To balance the solvent volatilization and the adhesive force between QD and substrate, ethanol is added into the TGA-QDs aqueous solution. Our study demonstrated the optimal volume ratio of water to ethanol is 2:1. Figure 1b displays the energy band structure of the photodetector. The QDs layer will harvest photons and then generate excitons (electron-hole pairs). The photogenerated excitons dissociate in the QD layer and then electrons transfer to the electron transport layer (ETL) while holes correspondingly to the hole transport layer (HTL). Figure 1c,d show the corresponding structure and scanning electron microscopy (SEM) cross-section image of the photodetector consisting of ETL (compact and mesoporous TiO_2_), active layer (TGA-QDs), HTL (Spiro-OMeTAD) and Ag electrode. Figure 1e shows the ultraviolet-visible (UV-vis) absorption and photoluminescence (PL) spectra of CdSeTe QDs. The absorption spectrum covers broad range from ultraviolet (UV) to near-infrared (NIR), which provides wide spectral response of the photodetector. Figure S1a shows the X-ray diffraction (XRD) pattern of CdSeTe QDs, in which there are two sharp diffraction peaks between the standard zinc blende CdTe (JCPDS Card No. 65-1081) and CdSe (JCPDS Card No. 19-0191). The transition electron microscopy (TEM) in Figure 1f displays the size uniformity of colloidal CdSeTe QDs. Figure S1b is the histogram of particle size distribution of the QDs, showing that the average diameter of the CdSeTe QDs is estimated to be 5 nm. The inset of high-resolution TEM (HRTEM) image exhibits a 0.37 nm lattice spacing according to the (111) plane of CdSe (lattice spacing: 0.351 nm) and CdTe (lattice spacing: 0.374 nm). These demonstrate that the QDs have good crystallinity.

**Figure 2:**
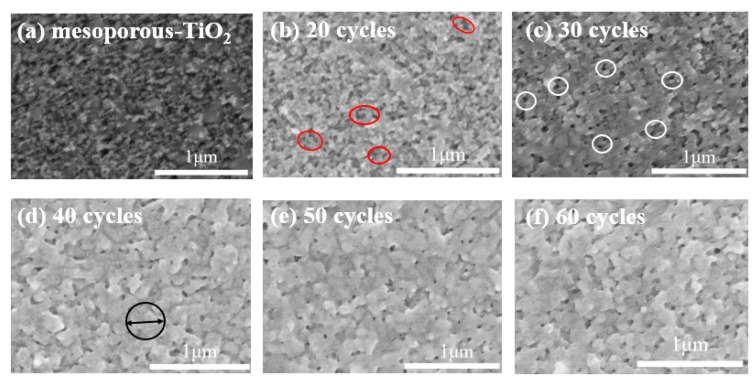
SEM images of QDs films deposited on mesoporous TiO_2_ substrate by spray coating with different deposition cycles: (**a**) mesoporous TiO_2_ substrate, (**b**) 20 cycles, (**c**) 30 cycles, (**d**) 40 cycles, (**e**) 50 cycles, (**f**) 60 cycles.

Figure 2 shows the SEM images of QDs films deposited by spray coating with different deposition cycles. The substrates are mesoporous TiO_2_ (m-TiO_2_) films as shown in Figure 2a. After 20 cycles of spray coating, the morphology of the m-TiO_2_ substrate cannot be completely covered by QDs as shown in Figure 2b (marked by red circles). The m-TiO_2_ substrate is completely covered by QDs after spray coating 30 cycles (Figure 2c). However, there are many pinholes (marked by white circles) in the QDs film. When the spray coating cycles are up to 40, the pinholes are diminished greatly (Figure 2d). The size of QDs aggregation is about 400 nm (marked by black circle). When the cycles of spray coating are more than 40, the morphology of the QDs film is no longer change. Figure S2a shows the UV-vis absorption spectra of QDs films fabricated by spray coating with different deposition cycles. The absorption value of the film gradually increases with increasing deposition cycles, indicating the increase of the thickness of the QD film. The fluctuations in the short wavelength of the absorption spectra are caused by the light scattering of TiO_2_ substrates. The gradual deepening of the color of the QD film in Figure S2b also represents the increase of thickness.

**Figure 3:**
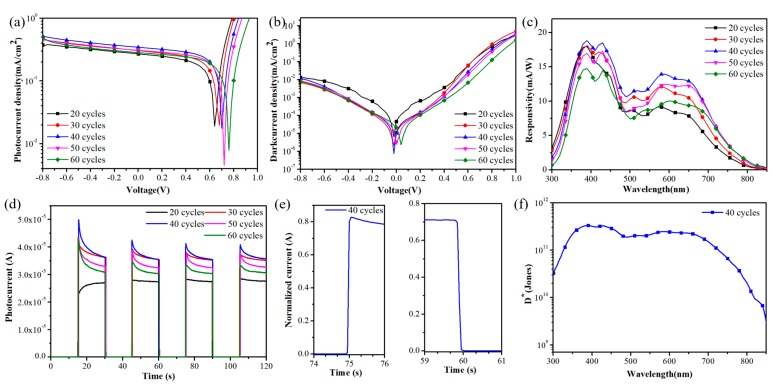
Current density-voltage curves of devices under (**a**) light and (**b**) dark conditions. (**c**) Spectroscopic response of the devices prepared by different spray coating cycles. (**d**) The current-time response of devices fabricated by different QD cycles under a light density of 100 mW/cm^2^. (**e**) The normalized current of the rise and decay time and (**f**) detectivity of the detector assembled by spray coating 40 cycles.

Figure 3a shows the current density-voltage curves of the photodetectors under light conditions. With the increase of spray coating cycles, the current density first increases and reaches the maximum when the deposition cycles are up to 40. It is attributed to both the increase of thickness and improvement of the coverage of QD films. When the deposition cycles are more than 40, the current density decreases gradually. The possible reason is that the film thickness exceeds the diffusion length of the charges, resulting in a decrease in the charge collection efficiency [22,23]. Figure 3b shows the current density-voltage curves of the devices under dark conditions. When the number of spray coating cycle is less than 30, the substrates cannot be completely covered by QDs. The much many pinholes of QD film caused serious carrier recombination, which results in high dark current density according to previous works [34,35]. When the substrates are uniformly covered by QDs, the devices show a lower dark current density due to the decrease of carrier recombination. However, when spray coating cycles are more than 40, the decrease of carrier separation efficiency and collection efficiency of QDs films result in the increase of the carrier recombination. Thus, the dark current density increases accordingly. The dark current density of the device is an indicator of noise degree of the photodetector, which reflects the ability to resist external interference. The lower dark current density means less background noise, indicating the better performance of the photodetector.

In Figure 3c the wavelength-dependent responsivity of photodetectors after depositing different number of spray cycles is shown. The responsivity (R) changes with the number of cycles is very similar to the photocurrent density of the devices. Initially the responsivity rises with the increase of the number of spray coating cycles until 40 cycles and starts dropping after that. Figure 3d exhibits current-time response of devices assembled by different spray coating cycles without bias voltage, representing the on-off switching properties of photodetectors. Although all variables show good on-off switching, the photodetector fabricated by spray coating 40 cycles shows the highest response current, which is consistent with its photocurrent density. However, the photocurrent of the QDs detector assembled by 20 cycles increases slowly to a steady value with the time under the exposer to the light, while the photocurrent of other devices increases rapidly and then drops to a stable value. Some previous studies have explained this behavior mainly by trap filling [14,36]. When the number of cycles is 20, the QD film cannot cover the TiO_2_ substrate completely, resulting in many traps in the film. The photogenerated carriers need to fill the traps first. This process takes a relatively long time, inducing a slow increase of the photocurrent. From 30 to 60 cycles, the coverage and quality of the film are improved, so there are fewer defects. Thus, the current quickly reaches the highest value. The following slight decrease can be related to possible photo-induced traps. Figure 3e shows the normalized current of the rise and decay of the detector assembled by 40 cycles. The rise time (T_r_) is defined as the time required to rise from 10% to 90% of the stable value of the current, while the decay time (T_d_) is defined as the opposite [37,38]. The T_r_ and T_d_ of the device assembled by 40 cycles are 0.04 s and 0.05 s, respectively, indicating a fast response.

Figure 3f shows the detectivity (D*) of the device fabricated by 40 cycles. The detectivity, which is a key parameter of photodetector performance, can be calculated as [39]

(1) $$D^{*}=\frac{R}{\sqrt{2qJ_{d}}}$$

where *R* is the responsivity, *q* is the elementary charge and *J_d_* is the dark current density [11,17]. As shown in Figure 3f, all the values of D* are more than 10^11^ Jones in the wavelength range of 350 nm to 700 nm. The relatively high D* indicates that the photodetector prepared by spray coating technology has good performance. We measured the external quantum efficiency (EQE) of the devices as shown in Figure S3. It is also found that the EQE of the device fabricated by 40 cycles has the highest EQE value (6%), which is consistent with the responsivity results.

**Figure 4:**
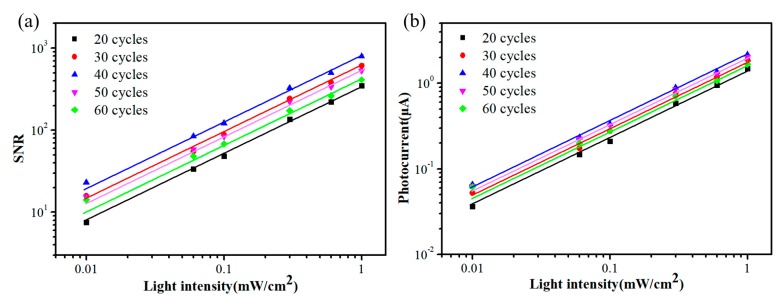
(**a**) Signal-to-noise ratio (SNR) and (**b**) linear dynamic range (LDR).

The signal-to-noise ratio (SNR) is the difference between the photocurrent minus the dark current, divided by the dark current, which represents the ratio of signal to background noise. A higher ratio indicates a lower disturbing noise. The linear dynamic range (LDR) is the parameter that the detector can detect the signal linearly within the range of incident light power, a larger LDR certifies a good detector function [14]. These detector characteristics are defined by the following formulas [3,40]

(2) $$\mathrm{SNR}=\frac{I_{light}-I_{dark}}{I_{dark}}$$

(3) $$\mathrm{LDR}=20\log\frac{J_{light}}{J_{dark}}$$

where *I_light_* and *I_dark_* are the photocurrent and dark current, respectively. *J_light_* is the photocurrent density under a light intensity of 1 mW/cm^2^ and *J_dark_* is the dark current density [41,42]. Figure 4a shows the SNR of the devices assembled by different cycles. It can be seen that the photodetector fabricated by 40 cycles has the highest SNR demonstrating remarkable noise endurance. LDR of devices is shown in Figure 4b. The LDR values of the device assembled by different QDs deposition cycles are 50.9 dB for 20 cycles, 55.7 dB for 30 cycles, 58.0 dB for 40 cycles, 54.5 dB for 50 cycles, 52.3 dB for 60 cycles, respectively. The detector prepared by 40 QDs deposition cycles has the largest SNR and LDR. This is consistent with the detection performance of the devices.

**Figure 5:**
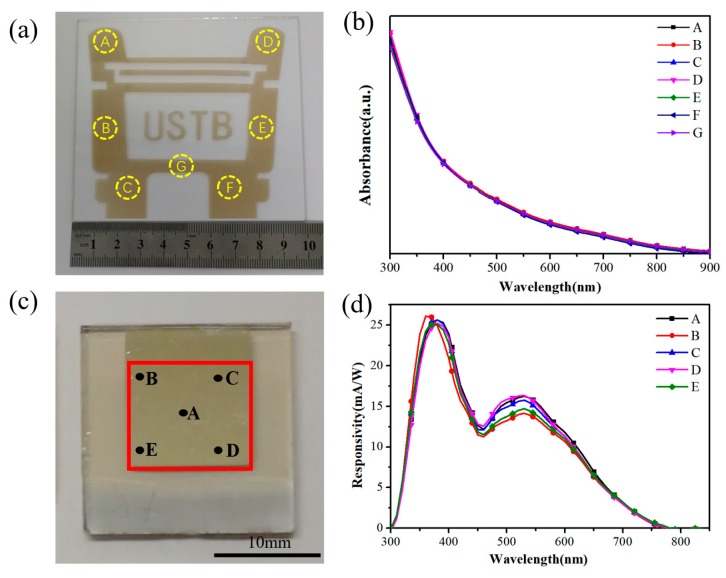
(**a**) The picture of the logo of QDs film deposited by spray coating with a size of 10 × 10 cm^2^ and (**b**) the UV-vis absorption spectrum at different positions in the logo film. (**c**) A large device with an active area of 1 cm^2^ and (**d**) the responsivity curves of the detector at different positions.

Figure 5a shows the pattern of QDs film with a large area of 10 × 10 cm^2^ deposited by spray coating. We selected seven points at different positions on the QDs film pattern (in Figure 5a) and the corresponding absorption spectra are shown in Figure 5b. These seven points have the same absorption value in the range of 300–900 nm which indicates the large QDs film prepared by spray coating has a good uniformity of thickness. Figure 5c shows the picture of a large size photodetector with an active area of 1 cm^2^ and we also selected five points in the active area of the device. The responsivity curves of these five points are as shown in Figure 5d. At 380 nm, the responsivity rates of these points are 25.6 mA/W, 24.9 mA/W, 25.6 mA/W, 25.2 mA/W, and 25.1 mA/W, respectively. At 530 nm, the responsivity rates of these points are 16.2 mA/W, 14.2 mA/W, 15.8 mA/W, 16.3 mA/W, and 14.7 mA/W, respectively. These five points have similar responsivity curves, which can be considered as a good uniformity QD distribution of the device with a large area. In addition, we compared two devices with different areas as shown in Figure S4. The active area of a device of normal laboratory-size is 0.1 cm^2^ while the larger one is 1 cm^2^. Figure S5 exhibits a serial of parameters of the detection performance of normal and large devices. The short circuit current density of small area and large area devices is 0.39 mA/ cm^2^ and 0.36 mA/ cm^2^, respectively. At 380 nm, the responsivity rates of two devices are 26.6 mA/W, 25.6 mA/W. At 550 nm, the responsivity rates of two devices are 16.8 mA/W, 15.5 mA/W. The rise time of the two devices is 0.04 s and 0.05 s, and the decay time is 0.07 s and 0.05 s, respectively. Figure S5d shows the statistical responsivity distribution for the different-size detectors at the wavelength of 380 nm and 550 nm. Although the responsivity of large-area devices is slightly lower than that of small-area devices, it still maintains a good level. These parameters indicate that the large-size device has the same detection capability as the conventional-size device, which also proves that the spray coating process is suitable for preparing large-area devices.

## 4. Conclusions

We exploited an efficient scalable spray coating process of CdSeTe QDs film for photodetectors. The mixed solvent of the QD solution was proposed to balance the solvent volatilization and the adhesive force of the QDs to the substrate so as to improve the quality of the thin film. When the number of cycles of the spray-coated quantum dots reached 40, the detector showed the best performance, with a response range of 300–800 nm, the responsivity of 15 mA/W, detectivity of more than 10^11^ Jones and rise time of 0.04 s. The large size (10 × 10 cm^2^) logo pattern QDs film prepared by spray coating displayed excellent uniformity of absorbance and thickness. In addition, it is found that the photodetector based on large-size QD film (the active area 1 cm^2^) has the same detection performance compared to the device based on typical laboratory-size QDs film (the active area 0.1 cm^2^), indicating that spray coating process has a broad application prospect for the realization of large-scale industrial production.

## Figures and Tables

**Table 1:** The main chemicals used in this article.

Chemicals	Reagent Purity	Manufacturer
selenium powder (Se)	99.0%	Alfa Aesar (shanghai, China)
cadmium oxide (CdO)	99.99%	Aladdin (shanghai, China)
tellurium powder (Te)	99.99%	Aladdin (shanghai, China)
paraffin liquid	99.0%	Aladdin (shanghai, China)
oleic acid (OA)	99.0%	Guoyao China (shanghai, China)
trioctylphosphine (TOP)	90.0%	Aladdin (shanghai, China)
oleylamine (OAm)	80–90%	Aladdin (shanghai, China)
mercapto acetic acid (TGA)	90.0%	Alfa Aesar (shanghai, China)

## Supplementary Materials

The following are available online at https://www.mdpi.com/2079-4991/9/12/1738/s1, Figure S1: (a) XRD pattern and (b) size distribution histogram of CdSeTe QDs, Figure S2: (a) The UV-vis absorption spectrum and (b) photograph of QDs films fabricated by spray coating with different deposition cycles, Figure S3. External quantum efficiency (EQE) spectra of photodetectors with different spray coating cycles, Figure S4. Contrast photograph of different sizes of photodetectors (the area circled in red is the active area), Figure S5. Current density-voltage curves of devices under light conditions. (b)The spectroscopic response of the devices of different sizes. (c) The normalized current of rise and decay time of different-size devices. (d) Statistical responsivity distribution for the different-size detectors at the wavelength of 380nm and 550nm.
