# Supplementary material for: Spray Coated Colloidal Quantum Dot Films for Broadband Photodetectors
Source: Nanomaterials (Basel). 2019 Dec 6;9(12):1738. doi: 10.3390/nano9121738 (PMC6955664; doi:10.3390/nano9121738)
Supplement: Supplementary file 1 [file nanomaterials-09-01738-s001.pdf]

## Supplementary materials

# Spray Coated Colloidal Quantum Dot Films for Broadband Photodetectors

**Kaixuan Song [1], Jifeng Yuan [1], Ting Shen [1], Jiuyao Du [1], Ruiqi Guo [1], Tönu Pullerits [2] and Jianjun Tian [1,*]**

[1] Institute for Advanced Materials and Technology, University of Science and Technology Beijing, Beijing 100083, China; (K.S. songkx1969931415@163.com); (J.Y. 13301203163@163.com); (T.S. shentingustb@163.com); (J.D. jiuyaodu@163.com); (R.G. ruiqiguo1217@163.com)

[2] Department of Chemical Physics and NanoLund, Lund University, Box 124, 22100 Lund, Sweden; tonu.pullerits@chemphys.lu.se

* Correspondence: tianjianjun@mater.ustb.edu.cn

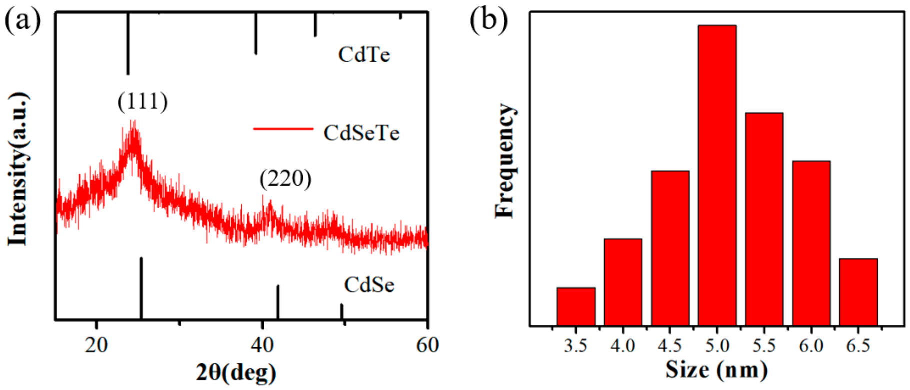


**Figure S1.** (a) XRD pattern and (b) size distribution histogram of CdSeTe QDs.

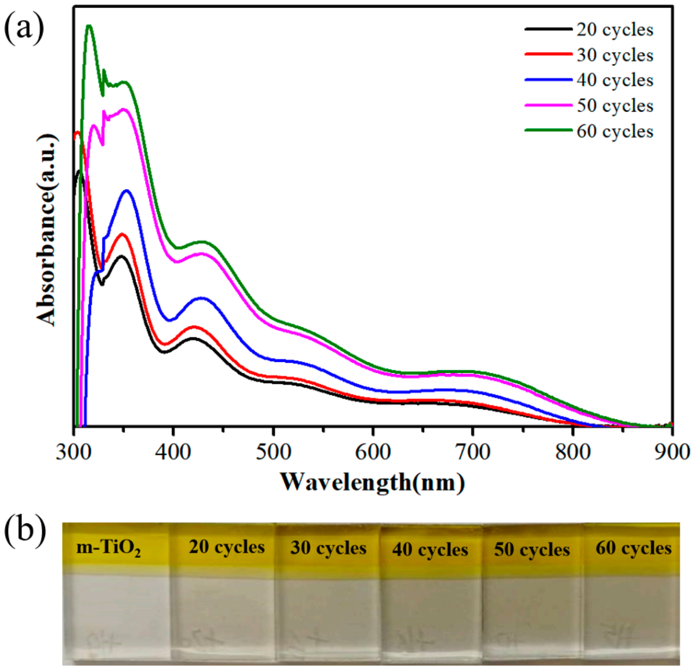


**Figure S2.** (a) The UV-vis absorption spectrum and (b) photograph of QDs films fabricated by spray coating with different deposition cycles.

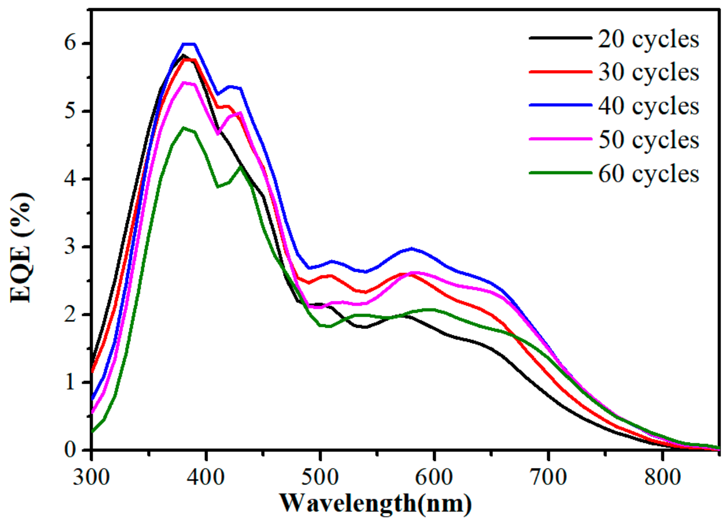


**Figure S3.** External quantum efficiency (EQE) spectra of photodetectors with different spray coating cycles.

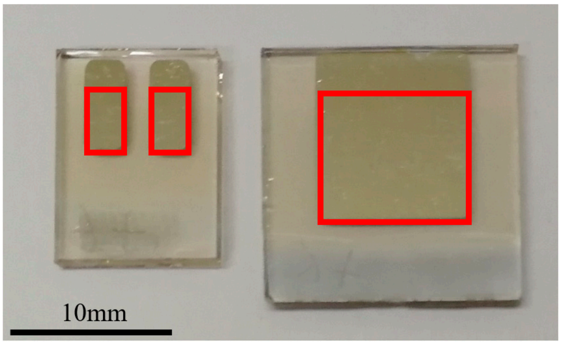


**Figure S4.** Contrast photograph of different sizes of photodetectors (the area circled in red is the active area).

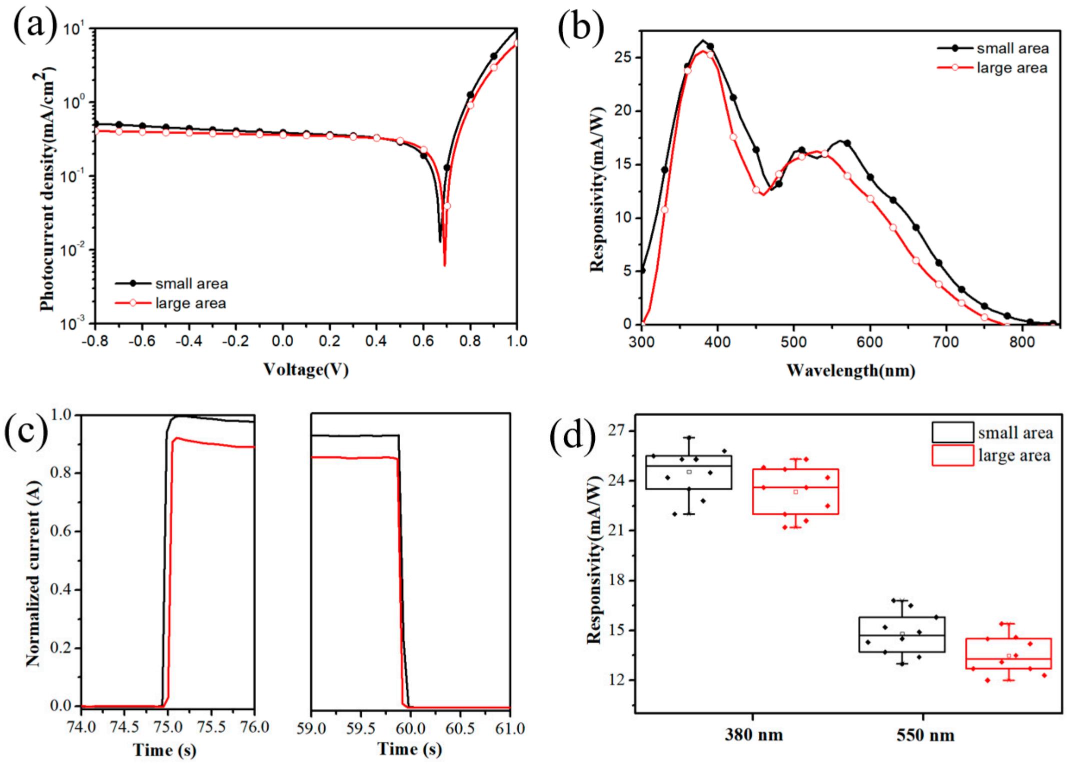


**Figure S5.** Current density-voltage curves of devices under light conditions. (b)The spectroscopic response of the devices of different sizes. (c) The normalized current of rise and decay time of different-size devices. (d) Statistical responsivity distribution for the different-size detectors at the wavelength of 380nm and 550nm.
